# Fc-fused IL-7 mobilizes long-term HSCs in a pro-B cell-dependent manner and synergizes with G-CSF and AMD3100

**DOI:** 10.1038/s41375-021-01274-6

**Published:** 2021-05-18

**Authors:** Sora Kim, Young-Min Kim, Hyekang Kim, Yeon-Woo Kang, Subin Park, Sang-In Yang, Donghoon Choi, Young Chul Sung, Seung-Woo Lee

**Affiliations:** 1 Department of Life Sciences, Pohang University of Science and Technology, Pohang, Republic of Korea; 2 Division of Integrative Biosciences and Biotechnology, Pohang University of Science and Technology, Pohang, Republic of Korea; 3 Genexine, Inc., Seongnam-si, Gyeonggi-do Republic of Korea; 4 Research Institute of NeoImmunetech, Co., ltd. Bio Open Innovation Center, Pohang, Republic of Korea

**Keywords:** Haematopoietic stem cells, Bone marrow transplantation

## To the Editor:

Hematopoietic stem cell (HSC) transplantation is a procedure that reestablishes hematopoietic function by infusing healthy HSCs into patients with severe hematopoietic disorders. Blood HSCs have successfully replaced traditional bone marrow (BM) transplants because of the non-invasive and safe collection process, as well as the favorable clinical outcomes following HSC transplantation [1]. Currently, recombinant human granulocyte colony-stimulating factor (rhG-CSF) is widely used to mobilize HSCs from the BM into the peripheral blood (PB) [2]. However, up to 40% mobilization failure has been reported in patients treated with G-CSF-based regimens, indicating a clinical need to develop successful therapies to induce sufficient stem cell mobilization [3].

Interestingly, it was reported that repeated administration of recombinant human interleukin-7 (rhIL-7) in mice mobilizes hematopoietic stem and progenitor cells (HSPCs) from the BM, with increases of lymphoid, myeloid, and erythroid cells in the periphery [4–6]. However, the cellular compartment of mobilized HSPCs in response to IL-7 and its underlying mechanisms remain unclear. Herein, we investigated IL-7-induced HSC mobilization using hybrid Fc-fused long-acting rhIL-7 (rhIL-7-hyFc) which has greater in vivo stability than conventional rhIL-7 [7].

**Fig. 1:**
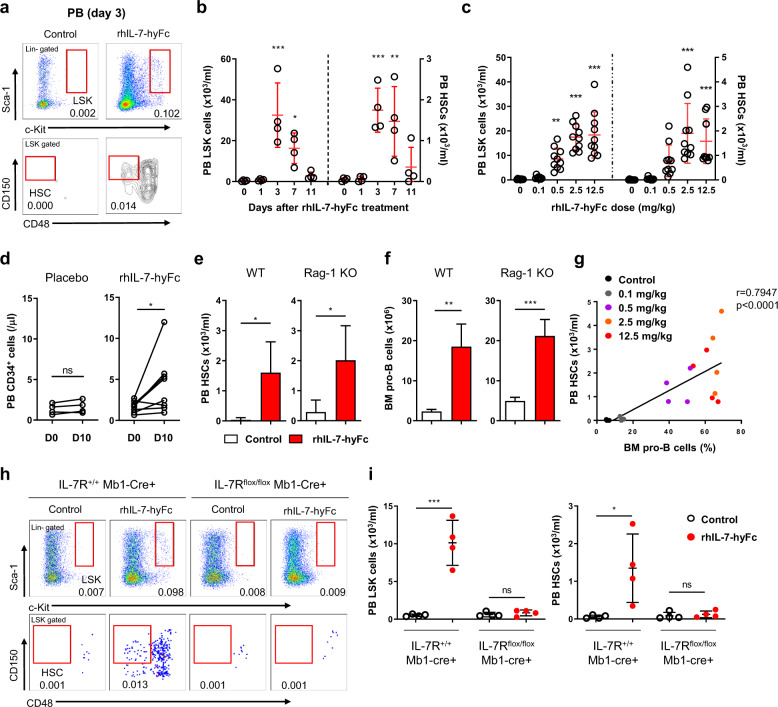
Single dose administration of rhIL-7-hyFc mobilizes long-term HSCs in a pro-B cell-dependent manner. **a**, **b** Kinetics of Lineage^−^Sca-1^+^c-Kit^+^ (LSK) cell and hematopoietic stem cell (HSC) mobilization upon rhIL-7-hyFc (2.5 mg/kg). Representative dot plot at day 3 post-treatment (**a**) and numbers of peripheral blood (PB) LSK cells and HSCs (**b**) (*n* = 4). **c** Dose-dependent mobilization of LSK cells and HSCs at day 3 post-treatment (0 and 0.1 mg/kg; *n* = 8, other groups; *n* = 10). **d** Blood CD34^+^ cells following the administration of single dose rhIL-7-hyFc (60 μg/kg) in healthy volunteers (placebo; *n* = 4, rhIL-7-hyFc; *n* = 8). **e** Numbers of PB HSCs mobilized by rhIL-7-hyFc treatment (2.5 mg/kg) in wild-type (WT) and recombination-activating gene 1 knockout (Rag-1 KO) mice (control; *n* = 4, rhIL-7-hyFc; *n* = 5). **f** Numbers of BM pro-B cells in WT and Rag-1 KO mice at day 3 post-rhIL-7-hyFc treatment (control; *n* = 4, rhIL-7-hyFc; *n* = 5). **g** Correlation between mobilized PB HSCs and BM pro-B cells at day 3 post-rhIL-7-hyFc treatment (*n* = 4). **h**, **i** Mobilization of LSK cells and HSCs by rhIL-7-hyFc treatment in IL-7R^flox/flox^ Mb1-Cre+ mice. Representative dot plot (**h**) and numbers of PB LSK cells and HSCs (**i**) (*n* = 4). Data are representative of two or three independent experiments and presented as mean ± SD. *P* values were determined by one-way ANOVA with Dunnett’s multiple comparison for (**b**), (**c**), Wilcoxon signed-rank test for (**d**), unpaired *t* test for (**e**), (**f**), (**i**), and Spearman’s correlation test for (**g**). “*n*” indicates the sample number. *P < 0.05, **P < 0.01, ***P < 0.001.

To identify which fractions of HSPCs are mobilized by exogenous IL-7, we treated rhIL-7-hyFc as a single dose into wild-type (WT) mice and analyzed HSCs and progenitor subsets (short-term HSC (ST-HSC), hematopoietic progenitor cell-2 (HPC-2), and multipotent progenitor (MPP)). Following rhIL-7-hyFc injection, an increase in circulating HSPCs (phenotyped by Lineage^−^Sca-1^+^c-Kit^+^, LSK) and a reduction in BM HSPCs were detected (Fig. 1a, Supplementary Fig. S1a, d, e), suggesting the mobilization of HSCs and progenitors from the BM into the periphery. HSC mobilization peaked at day 3 post-treatment and continued into day 7 in a dose-dependent manner (Fig. 1b, c, Supplementary Fig. S1b, c). HSCs mobilized by rhIL-7-hyFc treatment exhibited multipotency and long-term reconstituting activity (Supplementary Fig. S1f, g). Based on our animal study, we explored whether rhIL-7-hyFc-induced mobilization of HSCs occurs in humans. We analyzed human peripheral blood mononuclear cells (PBMCs) isolated from healthy volunteers after rhIL-7-hyFc administration [8]. Results showed a significant increase in PB CD34^+^ cells in the rhIL-7-hyFc-treated group compared with the placebo group (Fig. 1d, Supplementary Fig. S1h, i), demonstrating the ability of rhIL-7-hyFc to mobilize HSPCs in humans.

Given that most HSPCs do not express IL-7 receptor (IL-7R) whereas mature T cells and developing B-cells highly express IL-7R (Supplementary Fig. S2) [9], we expected that IL-7R-expressing lymphocytes in the BM may contribute to HSC mobilization. Similar HSC mobilization was observed in recombination-activating gene 1-deficient mice (Rag-1 KO) (Fig. 1e, Supplementary Fig. S3a), demonstrating that mature T cells and developing pre-B and immature B cells are not required for IL-7-induced HSC mobilization. Following rhIL-7-hyFc treatment, pro-B cells proliferated excessively in both WT and Rag-1 KO mice, whereas pre-pro-B cells were largely unaffected (Fig. 1f, Supplementary Fig. S3b). Furthermore, increases in BM pro-B cells showed a strong correlation with blood HSC in response to rhIL-7-hyFc (Fig. 1g), suggesting that pro-B cell responses to rhIL-7-hyFc play a role in HSC mobilization. Thus, we generated IL-7R^flox/flox^ Mb1-Cre+ mice in which the IL-7R-dependent proliferation of pro-B cells was impaired (Supplementary Fig. S3c) [10]. Contrary to IL-7R^+/+^ Mb1-Cre+ control mice, rhIL-7-hyFc treatment in IL-7R^flox/flox^ Mb1-Cre+ mice did not show increased HSPCs in the PB nor reduction of BM HSPCs (Fig. 1h, i, Supplementary Fig. S3d, e). Collectively, these results demonstrate that BM pro-B cells are essential mediators for IL-7-induced HSC mobilization. rhIL-7-hyFc treatment did not reduce expression of HSC retention-associated genes, such as *Cxcl12*, *Scf*, and *Vcam1*, in non-hematopoietic niche cells (Supplementary Fig. S4a). However, rhIL-7-hyFc notably reduced expressions of VLA-4, but not CXCR4 or c-Kit, in HSCs in a pro-B cell-dependent manner (Supplementary Fig. S4b, c).

**Fig. 2:**
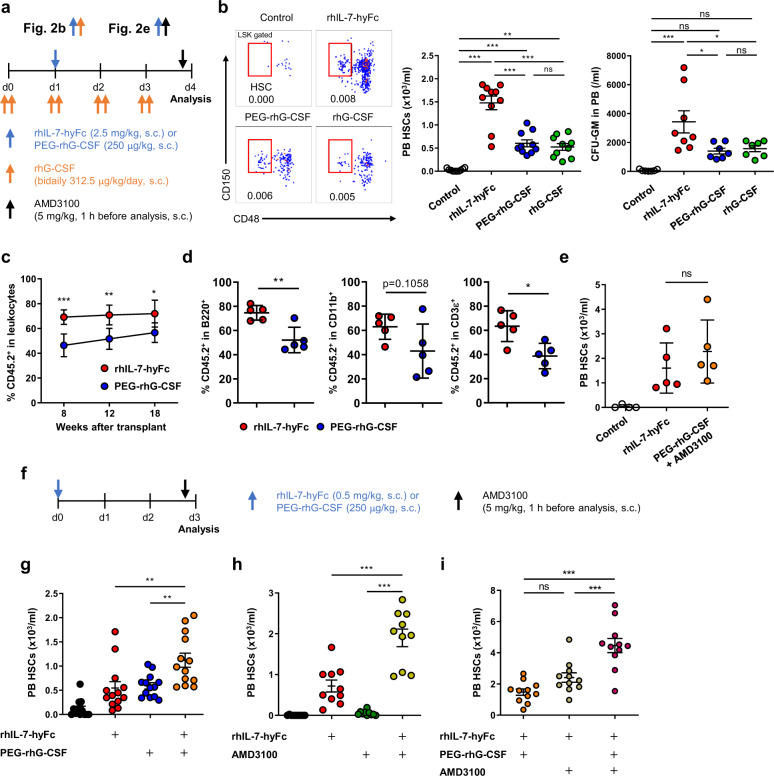
rhIL-7-hyFc has a superior function for HSC mobilization than G-CSF and synergizes with G-CSF and AMD3100. **a, b** Mobilization of HSCs by a single dose of rhIL-7-hyFc (2.5 mg/kg) or PEG-rhG-CSF (250 μg/kg), or a repeated dose of rhG-CSF (bidaily 312.5 μg/kg/day for 4 consecutive days). Experimental scheme (**a**). Representative dot plot and numbers of PB HSCs (*n* = 10) and CFU-GM (rhIL-7-hyFc; *n* = 8, other groups; *n* = 7) (**b**). **c, d** Competitive reconstituting assay with peripheral blood mononuclear cells (PBMCs) isolated from rhIL-7-hyFc or PEG-rhG-CSF-treated mice (CD45.2^+^) and BM competitor cells (CD45.1^+^). Blood chimerism in leukocytes at 8, 12, and 18 weeks (**c**) and in lineage cells at 8 weeks (**d**) (*n* = 5). **e** Mobilization of HSCs by control, 2.5 mg/kg rhIL-7-hyFc, or combination with 250 μg/kg PEG-rhG-CSF and 5 mg/kg AMD3100 (control; *n* = 4, other groups; *n* = 5). **f-i** Combined treatment with 0.5 mg/kg rhIL-7-hyFc, 250 μg/kg PEG-rhG-CSF, and 5 mg/kg AMD3100. Experimental scheme (**f**) and numbers of PB HSCs (**g–i**) (*n* = 13, 10, and 11 for **g**, **h**, and **i**, respectively). Data are pooled from two independent experiments (**b**, **g-i**, mean ± SEM) and are representative of 2 or 3 independent experiments (**c**–**e**, mean ± SD). *P* values were determined by one-way ANOVA with Tukey’s multiple comparison for (**b**), (**e**)**, (g-i)**, two-way ANOVA with Bonferroni’s multiple comparison for (**c**), and unpaired *t* test for (**d**). “*n*” indicates the sample number. *P < 0.05, **P < 0.01, ***P < 0.001.

We next sought to compare the efficacy of IL-7-induced mobilization to G-CSF, a standard agent used in clinics to mobilize HSCs [2]. Two types of G-CSF were used: a single dose of long-acting pegylated rhG-CSF (PEG-rhG-CSF), and repeated doses of rhG-CSF (Fig. 2a). All treatments downregulated BM cellularity but upregulated blood and splenic cell counts (Supplementary Fig. S5a, c, g). The single dose of rhIL-7-hyFc significantly increased blood HSPC and CFU-GM levels compared to those induced following PEG-rhG-CSF and rhG-CSF treatment (Fig. 2b, Supplementary Fig. S5b). Mobilized HSPCs by rhIL-7-hyFc were also detected in the spleen and their levels were slightly higher than that by G-CSF (Supplementary Fig. S5h-j). As previously observed in Supplementary Fig. S1a-c, rhIL-7-hyFc dropped HSPC and CFU-GM levels in the BM (Supplementary Fig. S5d-f). By contrast, G-CSF administrations reduced HSCs but maintained LSK cells in the BM, leading to CFU-GM counts comparable to control. This difference may result from the G-CSF-mediated HSPC expansion [11], which was not seen in rhIL-7-hyFc treatment. Because the mobilization efficacy of two G-CSF types was comparable (Fig. 2b, Supplementary Fig. S5b), we used long-acting PEG-rhG-CSF for subsequent experiments. We next evaluated the repopulating capacity of mobilized HSCs following rhIL-7-hyFc and PEG-rhG-CSF treatment, and observed that PBMCs isolated from rhIL-7-hyFc-treated mice had higher reconstituting activity, compared with PBMCs from PEG-rhG-CSF-treated mice (Fig. 2c, d). In clinical practice, G-CSF is often combined with AMD3100 to improve mobilization efficiency in patients who displayed insufficient mobilization with G-CSF treatment alone [3]. Interestingly, our data showed that single rhIL-7-hyFc treatment mobilized HSCs to levels similar to those after combined PEG-rhG-CSF and AMD3100 treatments (Fig. 2e), indicating a superior function of rhIL-7-hyFc compared to G-CSF.

To expand the clinical applicability of rhIL-7-hyFc, we evaluated whether the level of HSC mobilization by rhIL-7-hyFc could be enhanced with addition of G-CSF and AMD3100 (Fig. 2f). Injecting both rhIL-7-hyFc and PEG-rhG-CSF resulted in increased blood HSC concentrations compared to single treatments (Fig. 2g, Supplementary Fig. S6a). Combined administration of rhIL-7-hyFc and AMD3100 also showed a synergistic effect compared to rhIL-7-hyFc or AMD3100 alone (Fig. 2h, Supplementary Fig. S6c). Interestingly, the triple combination of rhIL-7-hyFc, PEG-rhG-CSF, and AMD3100 showed a further increase compared to the double treatments of rhIL-7-hyFc and PEG-rhG-CSF, or rhIL-7-hyFc and AMD3100 (Fig. 2i, Supplementary Fig. S6e). Collectively, our data showed that the combination of rhIL-7-hyFc with G-CSF and AMD3100 synergistically enhanced HSC mobilization. Consistent with previous report [12], G-CSF treatment reduced pro-B cell number in the BM (Supplementary Fig. S6b). However, the combination of rhIL-7-hyFc with G-CSF preserved pro-B cells in the BM, implying a rescue of B-lymphopoiesis (Supplementary Fig. S6b). rhIL-7-hyFc also upregulated pro-B cells in the BM along with AMD3100 treatment (Supplementary Fig. S6d).

In this study, we propose rhIL-7-hyFc (GX-I7, NT-I7, efineptakin-alfa) as a new agent that mobilizes HSCs from the BM into the PB after a single treatment. rhIL-7-hyFc promotes HSC mobilization indirectly since HSCs do not express IL-7R [9]. In addition, rhIL-7-hyFc does not regulate crucial interactions between HSCs and their niches, such as CXCR4/CXCL12 and c-Kit/SCF, which are the main targets of conventional mobilization agents [13]. Instead, our data suggests that IL-7R-expressing pro-B cells are critical for HSC mobilization. Interestingly, rhIL-7-hyFc treatment downregulated VLA-4 on BM HSCs in a pro-B cell-dependent manner. Since the VLA-4/VCAM-1 interaction is known to regulate HSC retention in the niche [14], IL-7-mediated reduction of VLA-4 may play a role in HSC mobilization. Notably, pro-B cells reside in perisinusoidal regions where HSCs co-locate, and express similar niche molecules such as CXCR4, c-Kit, and VLA-4 [10, 13, 15]. Thus, we are tempting to speculate the possibility that expanded pro-B cells by rhIL-7-hyFc may competitively occupy the niche, blocking retention interactions for HSCs to induce the mobilization.

Gene transcription profile revealed that a particular myeloid progenitor (PreGM) in the BM also expressed IL-7R (BloodSpot at www.bloodspot.eu). However, BM total myeloid progenitors (MPs), which contain PreGMs, did not express IL-7R in protein levels (Supplementary Fig. S2a) and showed a significant reduction after rhIL-7-hyFc treatment (Supplementary Fig. S1e), indicating MPs are not required to stem cell mobilization by rhIL-7-hyFc. IL-7 treatment is also known to indirectly promote myelopoiesis and erythropoiesis by induction of multiple cytokines such as GM-CSF, IL-3, and Flt3 ligand [6]. It leads us to hypothesize that expanding pro-B cells and other IL-7R-expressing cells, especially activated T cells, by rhIL-7-hyFc could express factors such as cytokines in the BM that may regulate HSC mobilization. Further studies remain to address those questions.

Phase 1 clinical study proved that rhIL-7-hyFc was safe and well-tolerated in healthy subjects (NCT02860715) [8]. Transient injection site reactions, which were resolved spontaneously, were reported but no serious adverse effects were observed. For the first time, we demonstrated an increase in CD34^+^ HSCs in the PB of healthy volunteers after rhIL-7-hyFc treatment. Injected dose of 60 μg/kg in humans (mouse equivalent dose: 0.75 mg/kg) is 3.3-fold lower than the optimal dose used in mice, so future clinical studies are needed to examine optimal and effective regimens. In addition, whether the mechanism by which IL-7 mobilizes BM HSCs in mice is similar to that in humans remains to be determined. Nonetheless, owing to the distinct mode of action to mobilize HSCs, our findings suggest rhIL-7-hyFc as a mobilizing agent and synergistic partner for G-CSF and AMD3100 in humans.

In conclusion, we demonstrated that a single dose administration of rhIL-7-hyFc mobilized multipotent long-term HSCs, mediated through IL-7R responses in BM pro-B cells in mice. rhIL-7-hyFc mobilized higher numbers of BM HSCs than G-CSF, and its efficacy was further augmented by co-treatments with G-CSF and AMD3100. Notably, a single rhIL-7-hyFc injection induced CD34^+^ stem cell mobilization in humans. Our results identify rhIL-7-hyFc as a promising agent for HSC mobilization, working in synergy with conventional mobilizing agents, G-CSF and AMD3100.

## Supplementary information

Supplementary information
