# Supplementary material for: Fc-fused IL-7 mobilizes long-term HSCs in a pro-B cell-dependent manner and synergizes with G-CSF and AMD3100
Source: Leukemia. 2021 May 18;35(10):3030–4. doi: 10.1038/s41375-021-01274-6 (PMC8478653; doi:10.1038/s41375-021-01274-6)
Supplement: Supplementary file 1 — Supplementary information [file 41375_2021_1274_MOESM1_ESM.pdf]

Supplementary information

Animal studies

All mouse strains were maintained in a specific pathogen-free animal facility at Pohang University of Science and Technology (POSTECH). C57BL/6, CD45.1 (#002014), Rag-1 KO (#002216), and IL-7R$^{flox/flox}$ (#022143) were purchased from Jackson Laboratories. Mb1-Cre mice were provided by Dr. Michael Reth (University of Freiburg, Germany) [1] and heterozygous Cre mice were crossed with IL-7R$^{flox/flox}$ mice (IL-7R$^{flox/flox}$ Mb1-Cre+) [2]. We used female or male (6-8 weeks) mice. All experiments were approved by the Institutional Animal Care and Use Committee of POSTECH.

Stem cell mobilization in mice

rhIL-7-hyFc (Genexine, GX-I7, NT-I7, efineptakin-alfa) was administrated subcutaneously as a single dose for mobilization study (up to 12.5 mg/kg) and combinational treatment (0.5 mg/kg), and blood was analyzed at the indicated times. For comparing the mobilization efficacy between rhIL-7-hyFc and G-CSF, we used a single dose of long-acting PEG-rhG-CSF and repeated doses of rhG-CSF. PEG-rhG-CSF (Amgen, Neulasta, 250 μg/kg) was administrated subcutaneously 3 days before harvest. rhG-CSF (Dong-A ST, Leucostim) was administrated subcutaneously at 312.5 μg/kg/day, twice a day, for 4 consecutive days, and blood was analyzed after the last injection. For evaluating the reconstituting activity or synergistic effect of rhIL-7-hyFc with G-CSF, we treated PEG-rhG-CSF as described above. Single-dose of AMD3100 (Sigma, 5 mg/kg) was injected subcutaneously 1 h before harvest.

Human sample analysis

The protocol was approved by the Institutional Review Board (IRB) of POSTECH (PIRB-2017-R071). Human peripheral blood mononuclear cell (PBMC) samples were obtained from healthy volunteers after rhIL-7-hyFc 60 μg/kg subcutaneously treatment with approval of the Institutional Review Board of Seoul National University Hospital (H-1605-099-762) after informed written consent in accordance with the Declaration of Helsinki (NCT02860715). PBMCs were further assessed by using a density gradient separation with Ficoll-Paque PLUS (GE Healthcare). Absolute $CD34^+$ cell count was calculated by multiplying the number of PBMCs (automated hematology analyzer) by the percentage of $CD34^+$ cells (flow cytometry) [3].

Cell preparation

Mice were anesthetized by intraperitoneal injection of ketamine (Yuhan Co.) and xylazine (Bayer) and blood was obtained by cardiac puncture. PBMCs were obtained by using a density gradient separation with Histopaque®-1083 (Sigma, 10831). Bone marrow (BM) cells were harvested by flushing femur and tibia with RPMI 1640 medium (WELGENE, LM 011-01) containing newborn calf serum (Gibco, 26010074). Spleen cells were obtained by mechanical dissociation through a 70 μm strainer (SPL Life Sciences Co., 93070). Absolute cell numbers were counted with an automated Vi-CELL XR analyzer (Beckman Coulter).

Transplantation

For evaluating the multipotency (Supplementary Fig. S1f), 2×10^6 BM cells from control mice ($CD45.2^+$) or PBMCs from 2.5 mg/kg rhIL-7-hyFc-treated mice ($CD45.2^+$) at day

3 post-treatment were transferred into lethally irradiated (9 Gy) recipients ($CD45.1^+$). Blood chimerism was analyzed 8 weeks after reconstitution.

For evaluating the long-term reconstituting activity (Supplementary Fig. S1g), serial transplantation was performed. PBMCs were isolated from the control or rhIL-7-hyFc (2.5 mg/kg)-treated mice ($CD45.2^+$) at day 3 post-treatment and 2×10^6 PBMCs were transferred into lethally irradiated recipients ($CD45.1^+$). After the immune reconstitution period for 16 weeks, freshly harvested BM cells from primary or secondary recipients (2×10^6) were transferred into lethally irradiated secondary or tertiary recipients ($CD45.1^+$), respectively.

For evaluating the competitive reconstituting activity (Fig. 2c, d), PBMCs were isolated from the rhIL-7-hyFc (2.5 mg/kg) or PEG-rhG-CSF (250 μg/kg)-treated mice ($CD45.2^+$) at day 3 post-treatment. Then, 2×10^6 PBMCs were transferred into lethally irradiated recipients ($CD45.1^+$/$CD45.2^+$) with freshly harvested competitor BM cells ($CD45.1^+$, 0.5×10^6 cells). Blood chimerism was analyzed 8, 12, and 18 weeks after reconstitution.

Flow cytometry

To analyze human $CD34^+$ cells in the blood, CD34 (BD, 348057, clone 8G12), CD45 (BD, 642275, clone 2D1), and 7-amino-actinomycin D (BD, 559925, 7-AAD) were used according to recommendations of the ISHAGE guidelines [3]. For analysis of mouse hematopoietic stem and progenitor cells (HSPCs) and myeloid progenitors (MPs), the following monoclonal antibodies were used: lineage cocktail (TER119 (ebioscience, 11-5921-82, clone TER-119), CD11b (ebioscience, 11-0112-82, clone M1/70), CD3ε (ebioscience, 11-0031-82, clone 145-2C11), B220 (ebioscience, 11-0452-82, clone RA3-6B2), CD19 (ebioscience, 11-0193-82, clone 1D3), NK1.1 (ebioscience, 11-5941-

82, clone PK136), Gr-1 (ebioscience, 11-5931-82, clone RB6-8C5), and MHCII (I-A/I-E, ebioscience, 11-5321-82, clone M5/114.15.2)), c-Kit (ebioscience, 25-1171-82, clone 2B8), Sca-1 (Biolegend, 108127, clone D7), CD150 (ebioscience, 46-1502-82, clone mShad150), CD48 (ebioscience, 17-0481-82, clone HM48-1), CD16/CD32 (ebioscience, 12-0161-82, clone 93), and CD34 (ebioscience, 11-0341-85, clone RAM34). For analysis of mouse B cell subsets, B220, CD43 (Biolegend, 143203, clone S11), CD24 (Biolegend, 101821, clone M1/69), IgM (ebioscience, 12-5890-82, clone eB121-15F9), and IgD (Biolegend, 405713, clone 11-26c.2a) were used. For the mechanistic study, CD127 (ebioscience, 12-1271-81, clone A7R34), CD45 (ebioscience, 17-0451-82, clone 30-F11), CXCR4 (Biolegend, 146507, clone L276F12), and VLA-4 (BD, 553157, clone R1-2) were additionally used. HSPCs and MPs were identified by the following phenotypic markers: LSK cells (Lin$^-$Sca-1$^+$c-Kit$^+$), HSCs (Lin$^-$Sca-1$^+$c-Kit$^+$CD150$^+$CD48$^-$), short-term HSCs (ST-HSCs; Lin$^-$Sca-1$^+$c-Kit$^+$CD150$^-$CD48$^-$), hematopoietic progenitor cells-2 (HPC-2; Lin$^-$Sca-1$^+$c-Kit$^+$CD150$^+$CD48$^+$), multipotent progenitors (MPPs; Lin$^-$Sca-1$^+$c-Kit$^+$CD150$^-$CD48$^+$), and MPs (Lin$^-$Sca-1$^-$c-Kit$^+$). B cell subsets were identified by the following phenotypic markers: pre-pro-B cells (B220$^+$CD43$^+$CD24$^-$), pro-B cells (B220$^+$CD43$^+$CD24$^+$), pre-B cells (B220$^+$CD43$^-$IgM$^-$IgD$^-$), immature B cells (B220$^+$CD43$^-$IgM$^+$IgD$^-$), and mature B cells (B220$^+$CD43$^-$IgM$^+$IgD$^+$). All samples were assessed on LSRFortessa or FACSCanto (BD) and analyzed using FlowJo software.

Quantitative real-time PCR

For isolation of BM CD45$^-$TER119$^-$7-AAD$^-$ cells, BM cells were harvested and remaining bones were crushed using a mortar and pestle. BM cells and bone

fragments were digested with dispase II (Gibco), Collagenase D (sigma), and DNaseI (sigma) for 1 hour at 37 ℃. After incubation, BM cells and bone fragments were passed through mesh filter and pooled. CD45$^-$TER119$^-$7-AAD$^-$ cells were sorted on MoFlo XDP (Beckman Coulter) and RNA was extracted using a TRIzol (Invitrogen). After genomic DNA elimination, cDNA was synthesized with a QuantiTect® Reverse Transcription Kit (Qiagen). Using Power SYBR Green PCR master mix (ThermoFisher scientific), real-time PCR was carried out on ViiA 7 Real-Time PCR system (ThermoFisher scientific). The relative expression of target genes was normalized to *Gapdh*. The primers were used as follows; *Gapdh* (forward: 5'-TGT GTC CGT CGT GGA TCT GA-3', reverse: 5'-TTG CTG TTG AAG TCG CAG GAG-3'), *Cxcl12* (forward: 5'-TTT CAG ATG CTT GAC GTT GG-3', reverse: 5'-GCG CTC TGC ATC AGT GAC-3'), *Scf* (known as *Kitl*, forward: 5'-CTC TTC AAC ATT AGG TCC CGA GAA AGG GAA AG-3', reverse: 5'-CTT CCA GTA TAA GGC TCC AAA AGC AAA GCC A-3'), and *Vcam1* (forward: 5'-TCG GGC GAA AAA TAG TCC TT-3', reverse: 5'-CCG GCA TAT ACG AGT GTG AA-3'). All procedures were performed according to the manufacturers' protocols.

Granulocyte-macrophage colony-forming unit (CFU-GM) assay

PBMCs, BM cells, and spleen cells were cultured at 37 ℃ for 10 days in MethoCult™ GF M3534 media (STEMCELL, 03534) containing recombinant cytokines, SCF, IL-3, and IL-6 [4].

Statistical analysis

The sample sizes of animal studies were chosen based on similar studies evaluating stem cell mobilization. Statistical methods to determine sample sizes were not performed. We did not carry out randomization or blinding for animal studies and did not exclude the data from analysis. Data were analyzed using GraphPad Prism version 8.4.1 for Windows (GraphPad Software, San Diego, California USA) and were presented as mean ± SEM (pooled data) or mean ± SD (representative data). For mouse studies, statistical significance was determined by unpaired two-tailed t-test for the comparison of two groups, one-way ANOVA with Dunnett's multiple comparison or Tukey's multiple comparison test and two-way ANOVA with Bonferroni's multiple comparison test for multiple comparisons, Log-rank test for survival comparison, and Spearman's correlation coefficient analysis for the correlation between BM pro-B cells and PB HSCs. For human studies, statistical significance was determined by Wilcoxon signed-rank test (two-tailed) for the comparison of two groups. P values < 0.05 were considered to be significant.

References

1. Hobeika E, Thiemann S, Storch B, Jumaa H, Nielsen PJ, Pelanda R*, et al.* Testing gene function early in the B cell lineage in mb1-cre mice. *Proc Natl Acad Sci U S A* 2006 Sep 12; **103**(37)**:** 13789-13794.

2. Fistonich C, Zehentmeier S, Bednarski JJ, Miao R, Schjerven H, Sleckman BP*, et al.* Cell circuits between B cell progenitors and IL-7(+) mesenchymal progenitor cells control B cell development. *J Exp Med* 2018 Oct 1; **215**(10)**:** 2586-2599.

3. Sutherland DR, Anderson L, Keeney M, Nayar R, Chin-Yee I. The ISHAGE guidelines for CD34+ cell determination by flow cytometry. International Society of Hematotherapy and Graft Engineering. *J Hematother* 1996 Jun; **5**(3)**:** 213-226.

4. May C, Rivella S, Chadburn A, Sadelain M. Successful treatment of murine beta-thalassemia intermedia by transfer of the human beta-globin gene. *Blood* 2002 Mar 15; **99**(6)**:** 1902-1908.

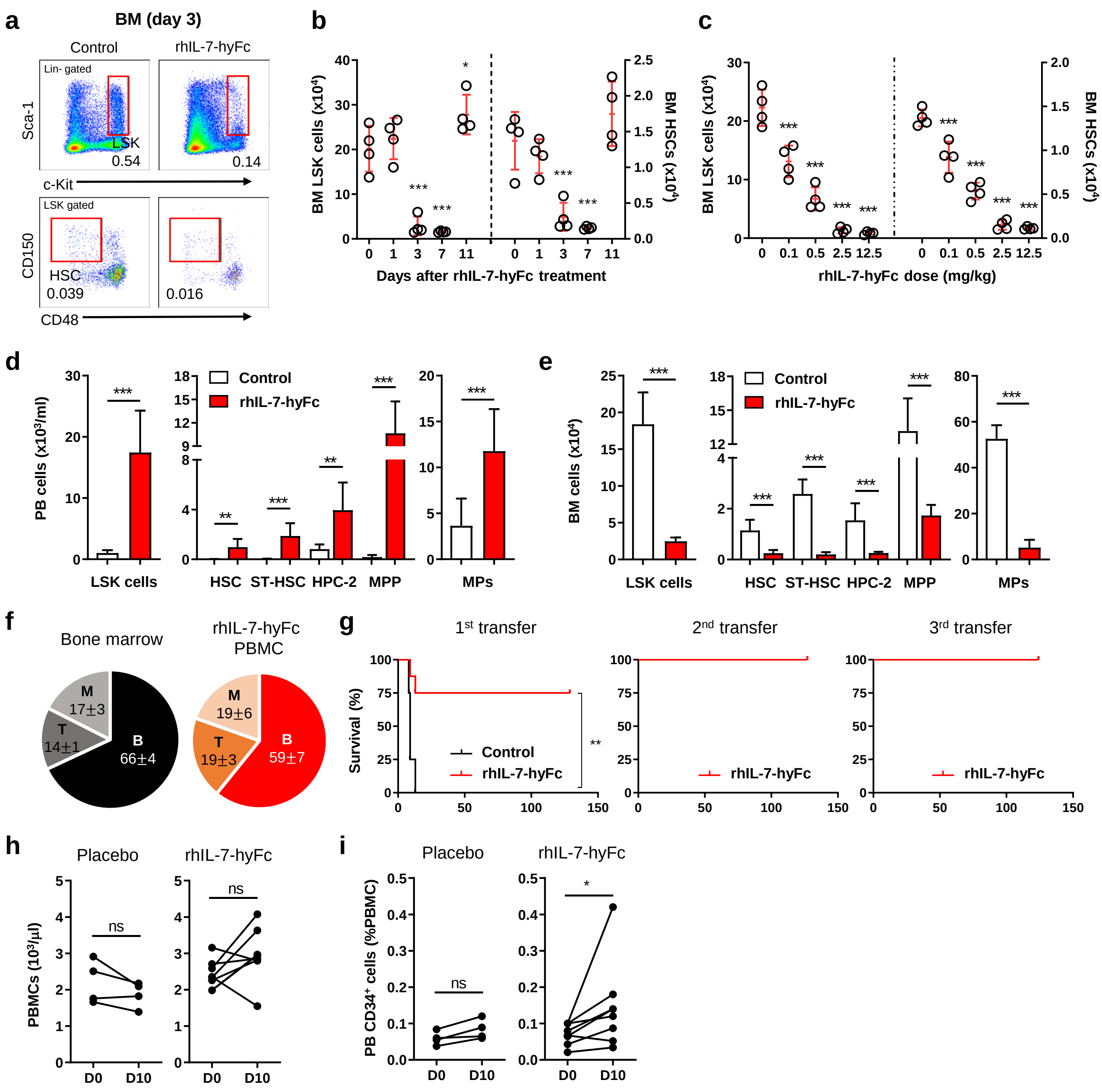

Supplementary Figure 1. Single dose administration of rhIL-7-hyFc induces the release of multipotent long-term HSCs from the BM. a, b Kinetics of BM LSK cells and HSCs upon rhIL-7-hyFc treatment (2.5 mg/kg). Representative dot plot at day 3 post-treatment (**a**) and numbers of BM LSK cells and HSCs (**b**) (*n* = 4). **c** Dose-dependent egress of BM LSK cells and HSCs at day 3 post-rhIL-7-hyFc treatment (*n* = 4). **d, e** Numbers of LSK cells, HSPC subsets (HSC, short-term HSC (ST-HSC), hematopoietic progenitor cell-2 (HPC-2), and multipotent progenitor (MPP) cells), and myeloid progenitors (MPs) in the blood (**d**) and BM (**e**) at day 3 post-rhIL-7-hyFc treatment (*n* = 8). **f** Multi-lineage reconstitution at 8 weeks in lethally irradiated mice transplanted with control BM cells or PBMCs isolated from rhIL-7-hyFc-treated mice. Blood $B220^+$ (B), $CD3\varepsilon^+$ (T), and $CD11b^+$ (M) cells were analyzed (BM; *n* = 5, rhIL-7-hyFc PBMC; *n* = 6). **g** Survival curve of serial transplantation with PBMCs from control or rhIL-7-hyFc-treated mice (control; *n* = 4, rhIL-7-hyFc; *n* = 8, 7, 6 for 1st, 2nd, 3rd transplantation, respectively). **h, i** Blood $CD34^+$ cells following the administration of rhIL-7-hyFc (60 μg/kg) in healthy volunteers. Total PBMC counts (**h**) and the percentage of $CD34^+$ cells in PBMCs (**i**) (placebo; *n* = 4, rhIL-7-hyFc; *n* = 8). Data are representative of two or three independent experiments (**a-g**, mean ± SD). *P* values were determined by one-way ANOVA with Dunnett's multiple comparison for **b, c**, unpaired t test for **d, e**, Log-rank test for **g**, and Wilcoxon signed-rank test for **h, i**. "*n* indicates the sample number. *$P < 0.05$, **$P < 0.01$, ***$P < 0.001$.

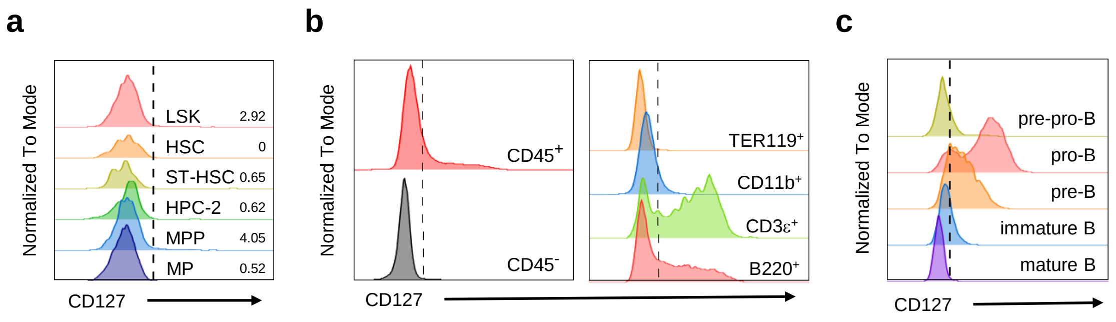

Supplementary Figure 2. IL-7R expression analysis. a-c Expression of CD127 on HSPCs and MPs (**a**), hematopoietic lineage cells (**b**), and B cell subsets (**c**) in the BM. Data are representative of three independent experiments and CD127-positivie cells were determined by isotype control.

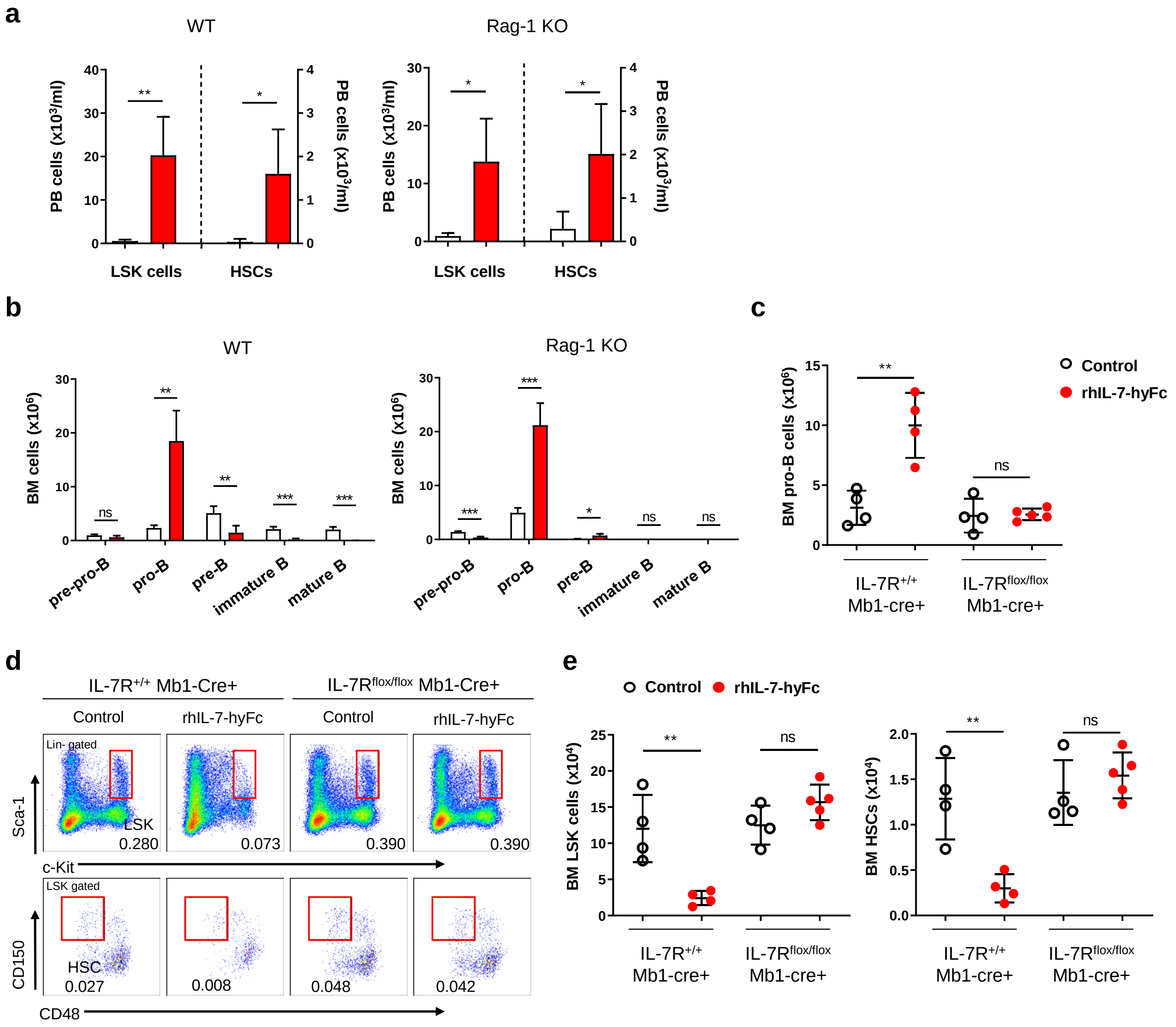

Supplementary Figure 3. pro-B-dependent mobilization of HSCs from the BM by rhIL-7-hyFc treatment. a, b Mobilization of LSK cells and HSCs by rhIL-7-hyFc treatment (2.5 mg/kg) in WT and Rag-1 KO mice. Numbers of PB LSK cells and HSCs (**a**) and numbers of BM B cell subsets (**b**) at day 3 post-treatment (control; n = 4, rhIL-7-hyFc; n = 5). **c** Numbers of BM pro-B cells at day 3 post-rhIL-7-hyFc treatment in WT and IL-7R$^{flox/flox}$ Mb1-Cre+ mice (rhIL-7-hyFc into IL-7R$^{flox/flox}$ Mb1-cre+; *n* =5, other groups; *n* = 4). **d, e** BM LSK cells and HSCs by rhIL-7-hyFc treatment (2.5 mg/kg) in IL-7R$^{flox/flox}$ Mb1-Cre+ mice. Representative dot plot (**d**) and numbers of BM LSK cells and HSCs (**e**) (rhIL-7-hyFc into IL-7R$^{flox/flox}$ Mb1-cre+; *n* =5, other groups; *n* = 4). Data are representative of two or three independent experiments and presented as mean ± SD. *P* values were determined by unpaired t test for **a-c, e**. “*n*” indicates the sample number. **P* < 0.05, ***P* < 0.01, ****P* < 0.001.

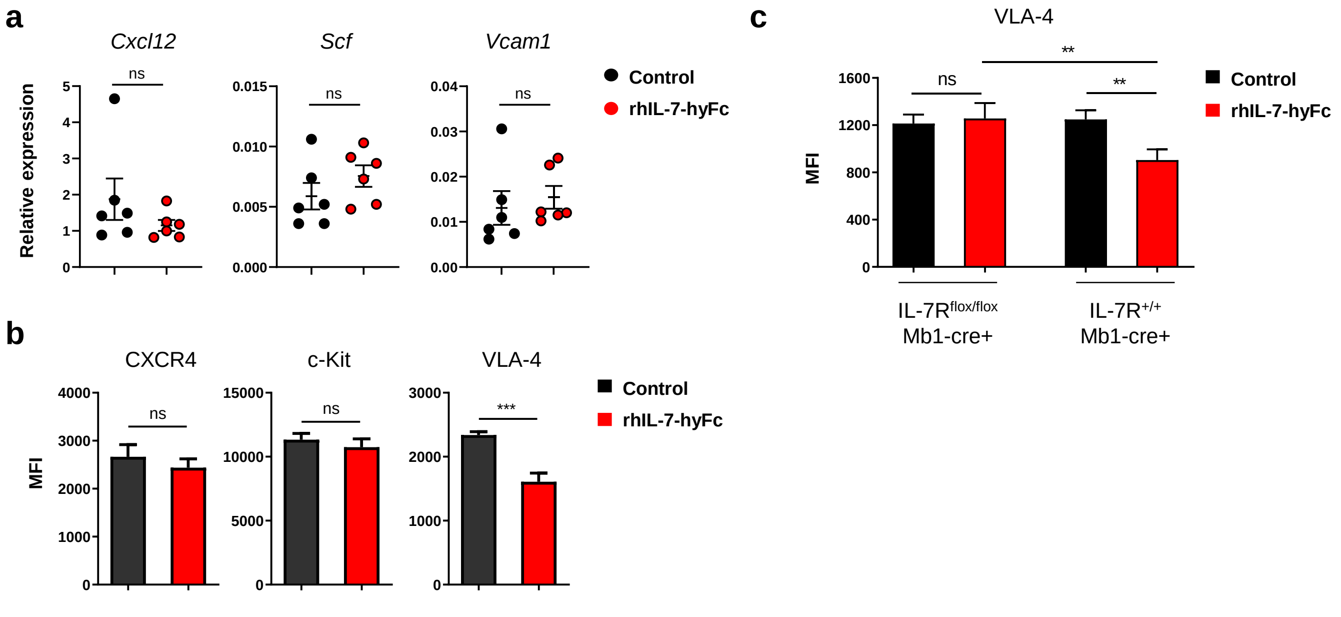

Supplementary Figure 4. Effect of rhIL-7-hyFc on niche factors. a-c Analysis of niche molecule expression in the BM at day 2 post-rhIL-7-hyFc treatment. Relative mRNA expression of genes related to stem cell retention on $CD45^{-}TER119^{-}$ non-hematopoietic cells (**a**) (*n* = 6). Median fluorescence intensity (MFI) of niche factors expressed on HSCs (**b**) (*n* = 4). MFI of VLA-4 expressed on BM HSCs in WT and IL-$7R^{flox/flox}$ Mb1-Cre+ mice (**c**) (control into IL-$7R^{flox/flox}$ Mb1-cre+; *n* =3, other groups; *n* = 4). Data are pooled from two independent experiments (**a**, mean ± SEM) and are representative of 2 or 3 independent experiments (**b**, **c**, mean ± SD). *P* values were determined by unpaired t test for **a, b** and one-way ANOVA with Tukey's multiple comparison for **c**. “*n*” indicates the sample number. $^{**}P < 0.01$, $^{***}P < 0.001$.

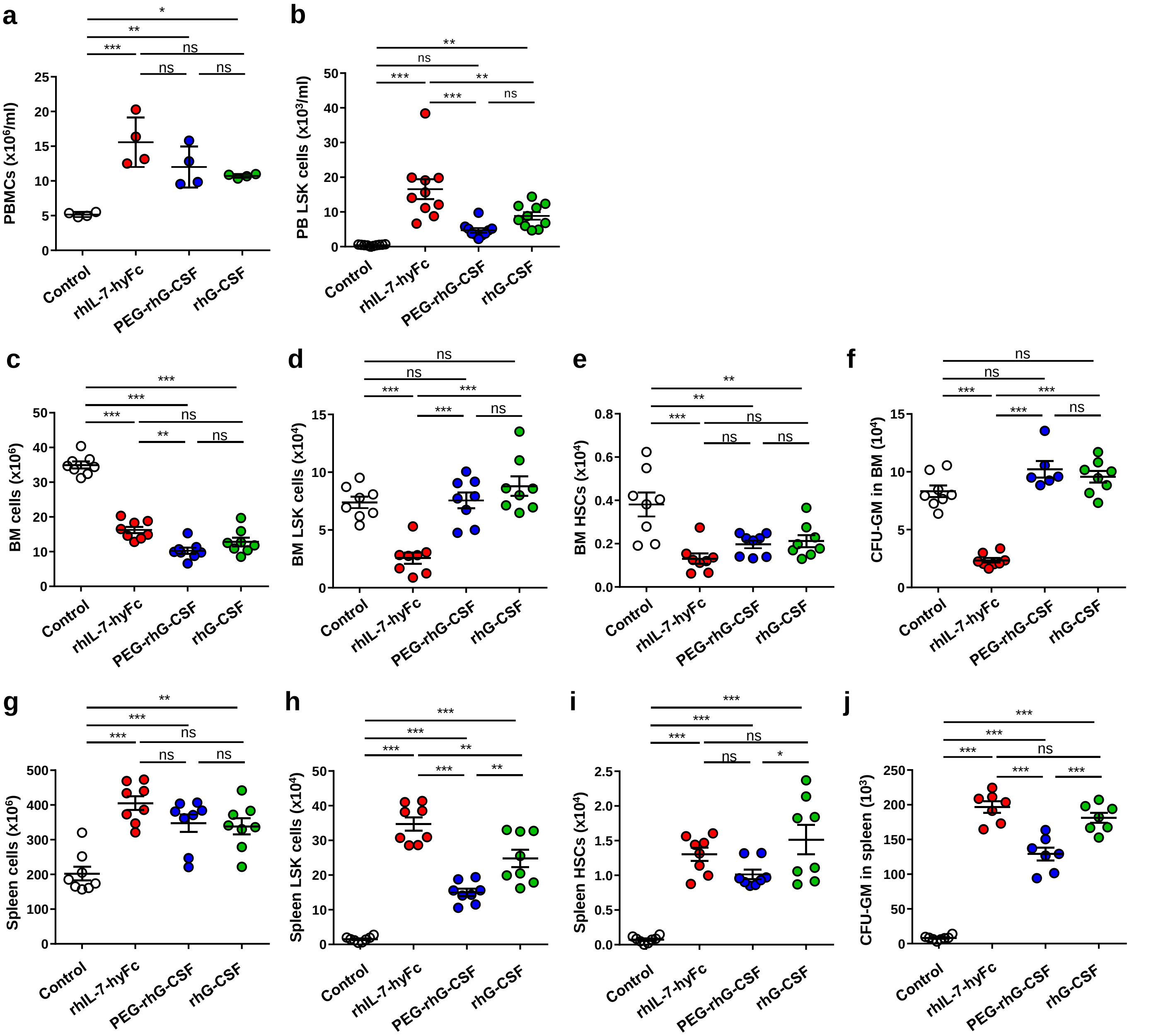

Supplementary Figure 5. rhIL-7-hyFc potently mobilizes HSPCs from the BM into the blood and accumulates HSPCs in the spleen. a, b Alteration of blood cells by rhIL-7-hyFc, PEG-rhG-CSF, or rhG-CSF treatment. Total PBMCs (**a**) (*n* = 4) and LSK cells in the blood (**b**) (*n* = 10). **c-f** Egress of BM LSK cells and HSCs by rhIL-7-hyFc, PEG-rhG-CSF, or rhG-CSF treatment. Total cellularity (**c**) (*n* = 8), LSK cells and HSCs (**d, e**) (*n* = 8), and CFU-GM (**f**) (PEG-rhG-CSF; *n* = 6, other groups; *n* = 8) in the BM. **g-j** Accumulation of spleen LSK cells and HSCs by rhIL-7-hyFc, PEG-rhG-CSF, or rhG-CSF treatment. Total cellularity (**g**) (*n* = 8), LSK cells and HSCs (**h, i**) (*n* = 8), and CFU-GM (**j**) (control; *n* = 8, other groups; *n* = 7) in the spleen. Data are representative of 3 independent experiments (**a**, mean ± SD) and are pooled from two independent experiments (**b-j**, mean ± SEM). *P* values were determined by one-way ANOVA with Tukey's multiple comparison for **a-j**. "*n*" indicates the sample number. *$P < 0.05$, **$P < 0.01$, ***$P < 0.001$.

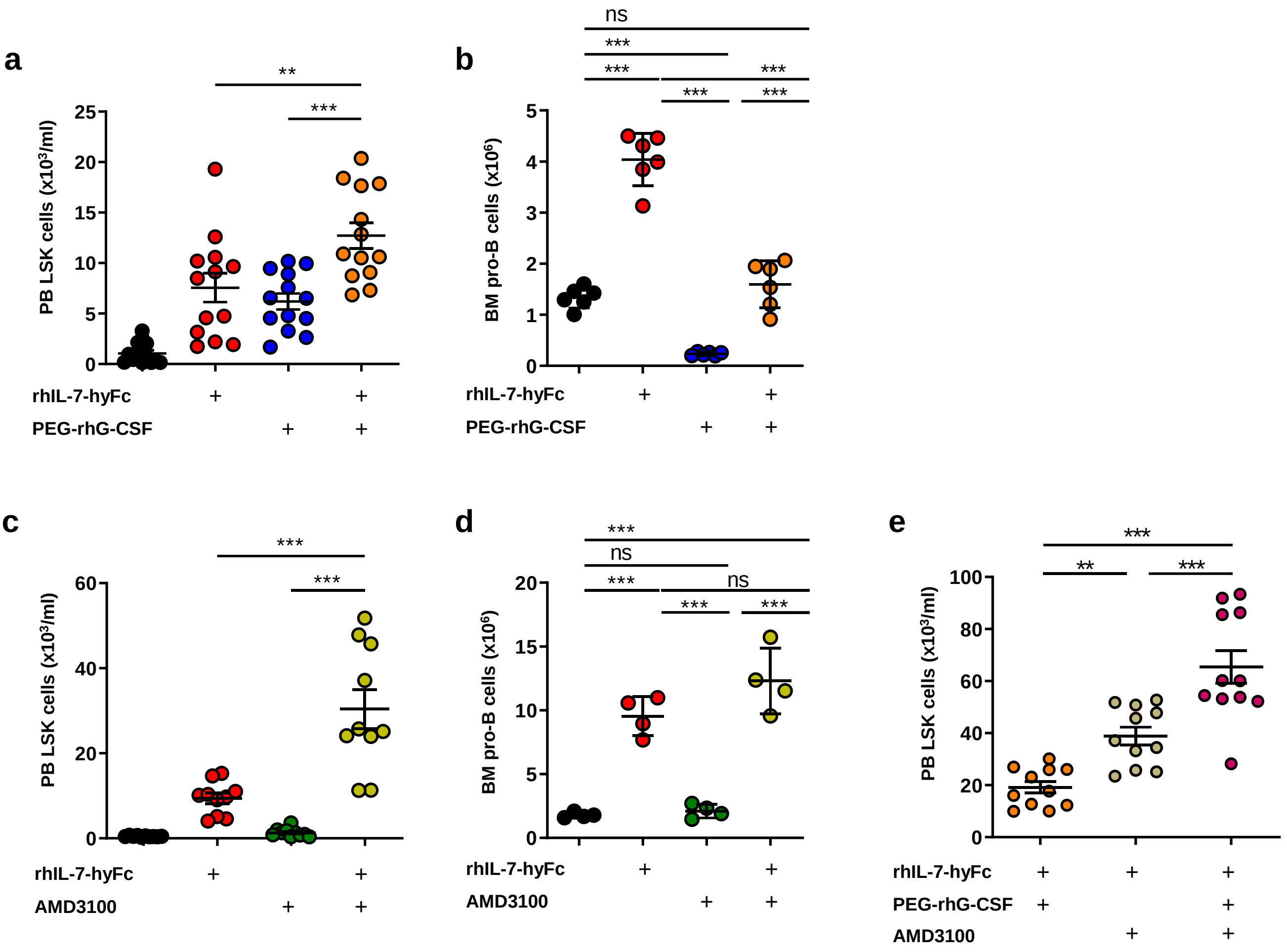

Supplementary Figure 6. Augmented mobilization of rhIL-7-hyFc with G-CSF and AMD3100. a, b Mobilization by combinational treatment with rhIL-7-hyFc and PEG-rhG-CSF. Numbers of PB LSK cells (**a**) (n = 13) and BM pro-B cells (**b**) (n = 6). **c, d** Mobilization by combinational treatment with rhIL-7-hyFc and AMD3100. Numbers of PB LSK cells (**c**) (n = 10) and BM pro-B cells (**d**) (n = 4). **e** Mobilization of LSK cells by triple combinational treatment with rhIL-7-hyFc, PEG-rhG-CSF, and AMD3100 (n = 11). Data are pooled from two independent experiments (**a**, **c**, **e**, mean ± SEM) and are representative of 3 independent experiments (**b**, **d**, mean ± SD). P values were determined by one-way ANOVA with Tukey's multiple comparison for **a-e**. "n" indicates the sample number. **$P < 0.01$, ***$P < 0.001$.
